# The impact of celebrity influence and national media coverage on users of an alcohol reduction app: a natural experiment

**DOI:** 10.1186/s12889-020-10011-0

**Published:** 2021-01-06

**Authors:** Claire Garnett, Olga Perski, Emma Beard, Susan Michie, Robert West, Jamie Brown

**Affiliations:** 1 Department of Behavioural Science and Health, University College London, London, WC1E 7HB UK; 2 Department of Clinical, Educational and Health Psychology, University College London, London, WC1E 6BT UK

**Keywords:** Smartphone app, Alcohol, Drinking, Engagement, User characteristics, Celebrity, Endorsement

## Abstract

**Background:**

Smartphone apps are increasingly used for health-related behaviour change and people discover apps through different sources. However, it is unclear whether users differ by mode of app discovery. *Drink Less* is an alcohol reduction app that received national media coverage in the UK caused by celebrity influence (a male TV and radio national broadcaster, aged 51). Our aim was to compare users who discovered the app before and after this coverage.

**Methods:**

A natural experiment assessing the impact of media coverage of *Drink Less* on users’ socio-demographic and drinking characteristics, app engagement levels, and extent of alcohol reduction. The study period was from 17th May 2017 to 23rd January 2019, with media coverage starting on 21st August 2018. Users were 18 years or over, based in the UK and interested in drinking less. Interrupted time series analyses using Generalised Additive Mixed Models were conducted for each outcome variable aggregated at the weekly level.

**Results:**

In 66 weeks prior to the media coverage, 8617 users downloaded the app and 18,959 in 23 weeks afterwards. There was a significant step-level increase in users’ mean age (B = 8.17, *p* < .001) and a decrease in the percentage of female users (B = -27.71, *p* < .001), though these effects dissipated non-linearly over time. No effect of media coverage was detected on employment type or on the percentage of at-risk drinkers, though the mean Alcohol Use Disorders Identification Test score was lower after the media coverage (B = -1.43, *p* = .031). There was a step-level increase in app engagement – number of sessions (B = 3.45, *p* = .038) and number of days used (B = 2.30, *p* = .005) – which continued to increase over time following quadratic trends.

**Conclusions:**

Celebrity influence leading to national media coverage in the UK of the *Drink Less* app was associated with more people downloading the app who were male, older and engaged with the app; and did not appear to impact employment inequality.

**Supplementary Information:**

The online version contains supplementary material available at 10.1186/s12889-020-10011-0.

## Background

Digital interventions, including smartphone applications (‘apps’), hold promise for supporting alcohol reduction [1, 2] though the reach of digital interventions remains limited in England [3]. People can discover apps by searching a commercial app store or online libraries of publicly endorsed health apps, or may hear about them from friends, family or social or mainstream media. Whilst there is research on who uses alcohol reduction apps, it is not known whether users differ according to how they discovered the app. *Drink Less* is an alcohol reduction app for people in the UK that received national media coverage in August 2018 following endorsement by a British male TV and radio presenter, aged 51 at the time, in a BBC (British public service broadcaster with international distribution) News article where people were encouraged to download and use the app [4] and in various other articles throughout the day*.* The interview was part of the publicity building up to the TV documentary “Adrian Chiles: Drinkers Like Me” that looked at the TV and radio presenter’s relationship with drinking and explored the public narrative around social and problem drinking (the documentary did not mention the app by name, only the interview to promote it). This provided the opportunity to conduct a natural experiment comparing the characteristics of users who discovered the app before and after this national media coverage of *Drink Less*.

Digital interventions have been found to be more effective at reducing alcohol consumption compared with no or minimal interventions [1, 2], and can deliver low-cost support in a timely manner to a large proportion of the population. Apps are one type of digital intervention that are increasingly affordable and prevalent in the UK population [5]. However, the evidence for alcohol reduction apps is inconclusive [6] with very few trials of digital interventions involving apps [1], which were limited by attrition and low engagement. Despite the potentially broad reach of apps, adoption in England is low with fewer than 5% of drinkers using any form of digital aid when making an attempt to reduce their consumption [3]. To improve the reach of apps, it is important to know who uses them and whether users differ by mode of discovery (e.g. searching an online app store such as Apple App Store or seeking recommendations from friends and family) [7]. The user characteristics of a number of alcohol reduction apps in the UK differ [8–11], though this may be due to different target audiences or marketing strategies. It is currently unclear whether users differ depending on how they discover the app, and whether this influences how they subsequently engage with it.

One of these smartphone apps, *Drink Less*, received celebrity endorsement and consequent coverage on national media including news articles and radio, allowing us to compare between users who discovered the app before and after the media coverage. *Drink Less* is an app, freely available in the UK, that was developed based on evidence and theory [12] to support people attempting to reduce their alcohol consumption, and has been evaluated in a factorial screening trial [9]. The content and development process of *Drink Less* are fully reported elsewhere [12]. Limited national promotion of the app occurred from May to December 2016 by organisations such as Cancer Research UK and a popular smoking cessation app, *Smoke Free*. From 2017, there was no media coverage of the app and all users discovered the app through searching on the app store or word-of-mouth. On the 21st August 2018, the app received national media coverage (e.g. the BBC News website, radio, television) as a result of positive interviews with the TV and radio presenter, Adrian Chiles. For example, on the BBC News website, Adrian Chiles was quoted as saying *“I encourage anyone, don’t judge yourself, don’t panic you’re not going to drop dead, but go on an app like ‘Drink Less’ and measure what you’re drinking, be honest with yourself for three weeks”* [4]*.* This resulted in a substantial spike in the number of downloads of *Drink Less*: the weekly average of 179 downloads between May 2017 and mid-August 2018 rose to 14,866 total downloads in the week commencing 20th August 2018. The app was consequently featured in the iTunes Top Health & Fitness apps chart and mentioned on radio interviews, which led to further media coverage through different platforms. This isolated period of celebrity influence and national media coverage provides the opportunity to conduct a natural experiment comparing the socio-demographic and drinking characteristics, engagement levels, and extent of alcohol reduction in users who discovered the app before and after the media coverage.

There are many health-related apps available for users to choose with little information about which are the most credible and/or effective. Celebrity influence on the uptake of apps may act to differentiate one particular app from others [13]. This positive effect that a celebrity can have on uptake is well-established in cancer screening [14, 15]. Following the public diagnosis of cervical cancer for Jade Goody, a young reality TV star, there was an increase in cervical screening attendance in England [16]. To the authors’ knowledge, this was the first study evaluating the impact of celebrity influence and consequent national media coverage on users of a health-related smartphone app. This study used an interrupted time series analysis to evaluate the immediate and longer-term effects of media coverage on *Drink Less* users’ socio-demographic and drinking characteristics, engagement levels, and extent of alcohol reduction.

## Methods

### Aim

The aim of this study was to investigate whether celebrity influence and consequent national media coverage of *Drink Less* had an effect on the characteristics of people using the app in terms of: a) socio-demographic and drinking characteristics; b) levels of engagement with the app; and c) extent of alcohol reduction.

### Design

A natural experiment assessing the effect of celebrity influence and media coverage of *Drink Less* on socio-demographic and drinking characteristics of users, engagement levels, and extent of alcohol reduction.

### Intervention

Users discovered *Drink Less* through app store search or browse, or word-of-mouth up until 21/8/2018 when there was celebrity influence and consequent national media coverage.

The media coverage of *Drink Less* began on 21/8/2018 as part of the publicity building up to the TV documentary “Adrian Chiles: Drinkers Like Me”, which was aired on 27/8/2018. The TV and radio presenter, Adrian Chiles, mentioned *Drink Less* in an interview that featured in a BBC News article that was the ‘most read’ BBC article on 21/8/2018 [4]. The interview was also reported by other mainstream national media platforms (e.g. The Times, Evening Standard, The Irish Post).

As well as news articles, the app was covered in the ‘related links’ for the TV documentary and mentioned on various radio shows (e.g. BBC Radio 5 Live interview with Dr. Claire Garnett on 21/8/2018; ‘Chiles on Friday’ on 24/8/2018). *Drink Less* was also featured on the iTunes Top Chart for Health & Fitness apps: sitting at #2 on 22/8/2018 and remaining in the Top Chart in subsequent weeks.

The study period was 89 weeks (between 17/5/2017 and 23/1/2019); a period during which there were no substantial changes to the app (see Supplementary Table 1 for an overview of minor app changes). The media coverage of *Drink Less* started on 21/8/2018 in the 67th week. Therefore, the study period was divided into pre- (66 weeks) and post-media coverage (23 weeks) segments.

### Sample

To be included, users had to be aged 18 years or above, based in the UK, responded that they were interested in drinking less when asked why they were using the app, had to have downloaded the *Drink Less* app between 17/5/2017 and 23/1/2019 (version 1.0.11 through to 1.0.16), and have agreed to the ‘Privacy Policy’ and ‘Terms and Conditions’ (available here: https://osf.io/akqy9/).

### Measures

Outcome variables were aggregated weekly with the measures for each user attributed to the week that the user downloaded the app (i.e. the post-media coverage period only included new users).

*Socio-demographic characteristics*, measured at baseline were: age (in years, continuous), sex (% female) and employment type (% non-manual). *Drinking characteristics*, measured at baseline, were: Alcohol Use Disorders Identification Test (AUDIT) score [17] (continuous) and the percentage of at-risk drinkers (% AUDIT> = 8).

*Engagement indicators*, measured over the 28 days following download, were: number of days used; number of sessions (a new session defined as a new screen view after 30 min of inactivity [18]); percentage of available screens viewed; and total time spent on the app in minutes (derived by calculating, for each user, the difference between the start and end time for each session and then summing across all sessions). These engagement measures were derived from raw screen view records, automatically collected by the app for each user, using a Python Framework, Pandas, within a Jupyter Notebook, an open-source web application. Another indicator of engagement was the follow-up response rate, measured as the percentage of users who had responded to the in-app survey delivered 28 days after app download.

*Extent of alcohol reduction* (in units, continuous) was measured as the change between baseline and 1-month follow-up in weekly alcohol consumption, derived from the quantity/frequency AUDIT questions at the 1-month follow-up. An intention-to-treat (ITT) approach was followed with non-responders assumed to be drinking at baseline levels.

### Analyses

Analyses were conducted in R studio. The analysis plan was pre-registered and the final dataset made available on the Open Science Framework (https://osf.io/w73ud/).

To estimate the impact of media coverage of *Drink Less* on each of the outcomes, separate interrupted time series analyses using Generalised Additive Mixed Models (GAMMs) [19] were conducted, at the weekly aggregated level, adjusted for month of the year and with seasonal smoothing terms fitted to account for seasonality.

All outcome variables were found to be normally distributed. This deviated from the analysis plan, which had assumed the engagement measures would fit a Poisson or quasi-Poisson distribution.

The regression models included the following terms:

$$ {y}_t={\beta}_0+{\beta}_1 trend+{\beta}_2{level}_t+{\beta}_3 slope+{e}_t, $$

where *β*_0_ is the baseline level for each outcome variable during the pre-media coverage period (i.e. intercept), *trend* is a variable coded 1 to 89 (number of weeks in the study period), which accounts for secular trends; *level*_*t*_ distinguishes the periods pre- and post-media coverage of *Drink Less*, coded 0 pre-media coverage and 1 post; and *slope* reflects the change in slope post-media coverage of *Drink Less*, coded 0 pre-media coverage and 1 to 23 thereafter (the number of weeks in the study period post-media coverage).

Plots of the autocorrelation function (ACF) and partial autocorrelation function (PACF) were checked for autocorrelation over time. The plots were used to identify plausible values for the autoregressive (AR) and moving average (MA) terms for the baseline model. Different models with various plausible AR and MA terms were compared with the baseline model using the Akaike Information Criterion (AIC), with smaller values indicating better model fit.

The fit of models including quadratic and cubic trends for the post-media coverage period were assessed using the AIC for each outcome variable (see Supplementary Table 2). The interpretation of the change in slope coefficients for the linear, quadratic (change in slope^2) and cubic (change in slope^3) trend models is detailed in Supplementary Table 3.

The analyses involving the drinking characteristics and engagement indicators were adjusted for socio-demographic characteristics. The analysis involving the extent of alcohol reduction was adjusted for baseline AUDIT scores [20].

## Results

Data were collected on 27,576 users of the *Drink Less* app. Table 1 gives descriptive statistics (mean, SD) for the aggregated data as a whole and as a function of pre- (*n* = 8617; weekly average of 131) and post- (*n* = 18,959; weekly average of 824) media coverage of *Drink Less*. See Supplementary Figure 1 for a graph showing the number of users downloading the app by week for the study period.

**Table 1:** Descriptives statistics for the aggregated data as a function of pre−/post-media coverage

Weekly aggregated variables	Whole time period (89 weeks), mean (SD)	Pre-media coverage (66 weeks), mean (SD)	Post-media coverage (23 weeks), mean (SD)
Mean age	42.4 (2.39)	41.4 (1.50)	45.6 (1.57)
Percentage female	55.0 (6.55)	56.5 (6.00)	50.7 (6.26)
Percentage non-manual employment	70.8 (4.28)	70.5 (4.27)	71.9 (4.20)
Mean AUDIT score	17.0 (1.22)	17.5 (0.96)	15.6 (0.66)
Percentage at-risk drinkers	92.4 (3.34)	93.4 (2.81)	89.4 (2.93)
Mean number of days used	9.8 (1.07)	9.5 (0.98)	10.5 (0.96)
Mean number of sessions	16.5 (1.91)	16.3 (1.95)	17.3 (1.60)
Percentage screens viewed	31.5 (1.56)	32.0 (1.39)	30.1 (1.06)
Mean time on app in minutes	41.8 (6.16)	42.2 (6.70)	40.6 (4.09)
Percentage follow-up response rate	12.6 (3.08)	12.6 (3.25)	12.7 (2.63)
Mean reduction in weekly alcohol consumption^a^	1.7 (0.87)	1.8 (0.97)	1.7 (0.45)

^a^intention to treat approach with non-responders assumed to be drinking at baseline levels

Table 2 reports the results for the adjusted, best fitting model for each outcome variable. Supplementary Table 4 reports the unadjusted, best fitting models for the engagement and drinking characteristics. Supplementary Table 5 reports the linear trend models for each outcome variable, including unadjusted models.

**Table 2:** Results of the best fitting model for each outcome variable (accounting for seasonality and autocorrelation)

	B (95% CI)	p
**Age,** cubic model		
Trend	0.03 (0.01, 0.05)	.003
Change in slope	−1.53 (−2.48, −0.58)	.002
Change in slope^2^	0.12 (0.03, 0.21)	.011
Change in slope^3^	−0.003 (−0.01, −0.001)	.019
Level	8.17 (5.47, 10.87)	<.001
**Sex (% female),** cubic model		
Trend	−0.04 (−0.07, −0.01)	.015
Change in slope	7.54 (3.30, 11.77)	.001
Change in slope^2^	−0.69 (−1.09, −0.28)	.001
Change in slope^3^	0.02 (0.01, 0.03)	.002
Level	−27.71 (−36.54, −12.89)	<.001
**Employment type (% non-manual),** linear model		
Trend	−0.01 (−0.06, 0.04)	.712
Change in slope	−0.11 (−0.34, 0.13)	.387
Level	3.14 (−0.54, 6.82)	.098
**AUDIT score**^**a**^, quadratic model		
Trend	−0.02 (−0.02, −0.01)	.006
Change in slope	0.21 (0.01, 0.41)	.045
Change in slope^2^	−0.06 (−0.01, 0.002)	.154
Level	−1.43 (−2.70, −0.16)	.031
**Percentage at-risk drinkers**^**a**^, cubic model		
Trend	0.003 (−0.04, 0.04)	.864
Change in slope	0.63 (−1.53, 2.78)	.571
Change in slope^2^	−0.11 (−0.31, 0.10)	.308
Change in slope^3^	0.004 (−0.002, 0.01)	.169
Level	−2.54 (−9.55, 4.47)	.480
**Number of days used the app**^**a**^, quadratic model		
Trend	0.01 (−0.01, 0.02)	.445
Change in slope	−0.42 (−0.67, −0.17)	.002
Change in slope^2^	0.02 (0.01, 0.03)	.002
Level	2.30 (0.73, 3.88)	.005
**Number of sessions on the app**^**a**^, quadratic model		
Trend	0.004 (−0.03, 0.03)	.801
Change in slope	−0.67 (−1.21, −0.13)	.017
Change in slope^2^	0.03 (0.01, 0.05)	.016
Level	3.45 (0.25, 6.65)	.038
**Percentage of screens viewed**^**a**^, cubic model		
Trend	−0.02 (−0.04, −0.01)	.004
Change in slope	0.31 (−0.57, 1.18)	.495
Change in slope^2^	−0.05 (−0.13, 0.03)	.233
Change in slope^3^	0.002 (−0.0004, 0.004)	.124
Level	−0.68 (−3.68, 2.31)	.657
**Time on app**^**a**^, linear model		
Trend	0.004 (−0.04, 0.05)	.849
Change in slope	−0.03 (−0.35, 0.29)	.833
Level	−4.14 (−9.15, 0.88)	.110
**Percentage follow-up response**^**a**^, linear model		
Trend	0.02 (−0.03, 0.06)	.449
Change in slope	−0.10 (−0.32, 0.11)	.356
Level	−1.57 (−5.34, 2.21)	.418
**Reduction in weekly alcohol consumption (ITT)**^**b**^, linear model		
Trend	0.004 (−0.003, 0.01)	.288
Change in slope	−0.02 (−0.06, 0.02)	.374
Level	0.23 (−0.43, 0.89)	.489

^a^adjusted for socio-demographic characteristics

^b^adjusted for baseline AUDIT scores

Change in slope^2^: quadratic trend

Change in slope^3^: cubic trend

### Socio-demographic characteristics

**Fig. 1:**
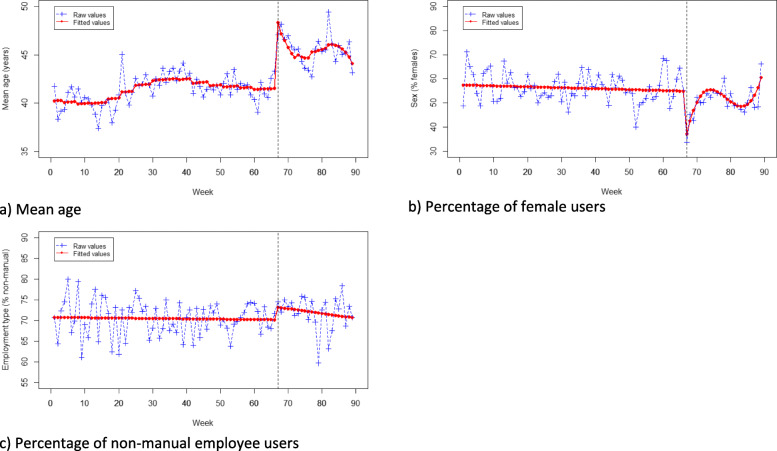
Plot of raw and fitted values for socio-demographic characteristics of users over time; **a** Mean age; **b** Percentage of female users; **c** Percentage of non-manual employee users

There was a step-level increase in the mean age of *Drink Less* users after the media coverage (B = 8.17, *p* < .001). The pre- and post-media coverage trends for age were significantly different (see Fig. 1a). The change in slope post-media coverage followed a cubic trend such that there was initially an accelerating decrease, followed by a short period of further incline and then an accelerating decrease.

Following media coverage of *Drink Less,* there was a significant step-level decrease in the percentage of female users (B = -27.71, *p* < .001). The pre- and post-media coverage trends for sex were significantly different (see Fig. 1b). The change in slope post-media coverage periods for sex followed a cubic trend, such that there was initially an accelerating increase towards pre-media coverage levels, followed by a short period of further decline and then an accelerating increase.

There was no significant step-level change detected in employment type following media coverage of *Drink Less* and no significant change in the slope between pre- and post-trends (see Fig. 1c)*.*

### Drinking characteristics

**Fig. 2:**
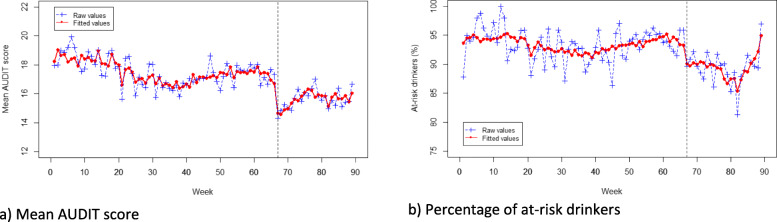
Plot of raw and fitted values for drinking characteristics of users over time; **a** Mean AUDIT score; **b** Percentage of at-risk drinkers

Following the media coverage of *Drink Less* there was a significant step-level decline in the mean AUDIT scores of users (B = -1.43, *p* = .031, see Fig. 2a). The post-media coverage period for AUDIT scores followed a quadratic trend, such that there was initially an accelerating increase, followed by a decline in mean AUDIT scores. The pre- and post-media coverage trends were significantly different for users’ mean AUDIT scores.

There was no significant step-level change in the percentage of at-risk drinkers after media coverage of *Drink Less* was detected (see Fig. 2b) and no significant difference between the pre- and post-media coverage trends.

### Levels of engagement with the app

**Fig. 3:**
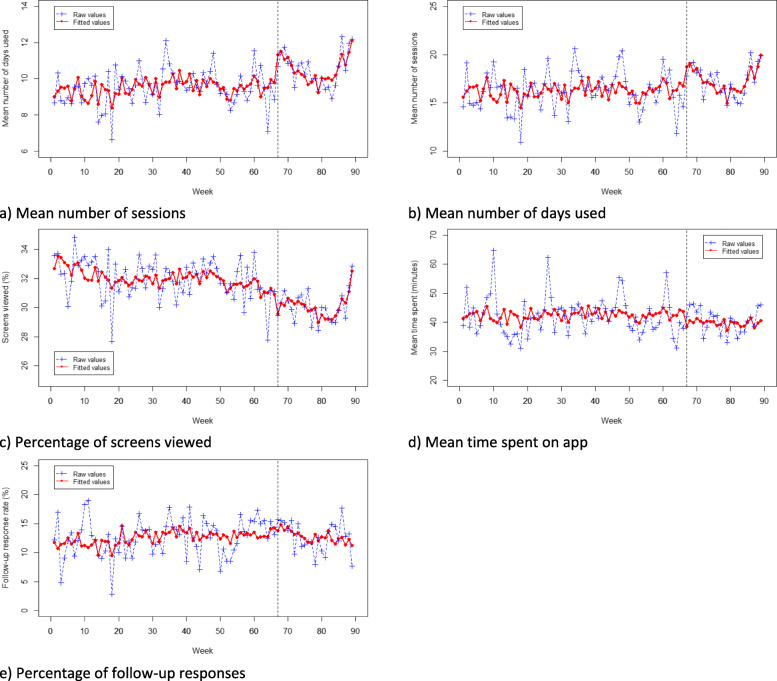
Plot of raw and fitted values for levels of engagement with the app over time; **a** Mean number of sessions; **b** Mean number of days used; **c** Percentage of screens viewed; **d** Mean time spent on app; **e** Percentage of follow-up responses

Following media coverage of *Drink Less* there was a significant step-level increase in the number of login sessions (B = 3.45, *p* = .038, see Fig. 3a) and in the number of days the app was used in the 28 days following download (B = 2.30, *p* = .005, see Fig. 3b). The pre- and post-media coverage trends were significantly different for the number of sessions and days used. The post-media coverage periods for both number of sessions and number of days using the app followed a quadratic trend, such that there was initially an accelerating decrease, followed by an incline in the number of sessions.

No significant step-level change following media coverage of *Drink Less* was detected for the percentage of screens viewed (see Fig. 3c), time spent on the app (see Fig. 3d), or follow-up response rate (see Fig. 3e). No significant differences were detected between the pre- and post-media coverage trends for percentage of screens viewed, time spent on app, or follow-up response rates.

### Extent of reduction in weekly alcohol consumption at 1-month follow-up

**Fig. 4:**
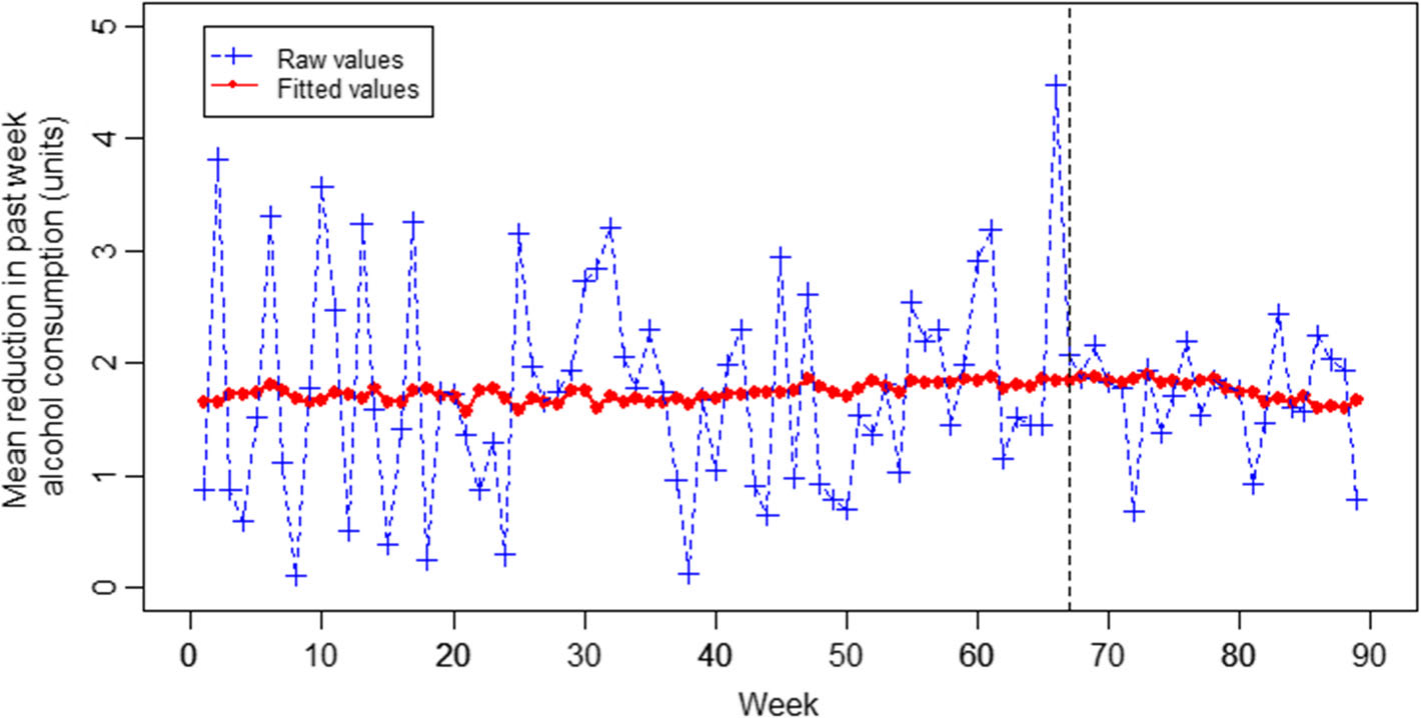
Plot of raw and fitted values for mean reduction in weekly alcohol consumption over time

Following media coverage of *Drink Less*, no significant step-level change was detected in the extent of reduction in weekly alcohol consumption at 1-month follow-up. No significant difference between post and the pre-media coverage trend was detected (see Fig. 4).

## Discussion

### Summary of findings

Celebrity influence leading to national media coverage of the *Drink Less* app was associated with a step-level increase in people downloading the app who were older and male. This immediate effect on the mean age and percentage of male users was not sustained in the long-term as it dissipated over time since the media coverage in a non-linear manner. No step-level change was detected in the proportion of users in non-manual employment. There was no change detected in the percentage of at-risk drinkers downloading the app, though there was a significant decrease in the mean AUDIT score of *Drink Less* users after the media coverage. This suggests that the media coverage of *Drink Less* may have encouraged people who were still at-risk drinkers but at the lower end of the scale to download the app. National media coverage had a positive effect on engagement with the app; there was a step-level increase in the number of sessions and days the app was used, which continued to increase over time following a quadratic trend. No effect was detected of the media coverage for other indicators of engagement (i.e. percentage of screens viewed, time on app or follow-up response rates) or for the reduction in weekly alcohol consumption at the 1-month follow-up.

### Implications

The results of this study indicate that celebrity influence and consequent national media coverage can have a substantial impact on who uses an app and how they engage with it. This has important implications for how digital interventions are promoted. In this study, the influence of Adrian Chiles – a male TV and radio presenter, aged 51 years at the time – resulted in an immediate increase in the proportion of males and the mean age of users, suggesting that people identifying with Adrian Chiles were more likely to download *Drink Less*. This effect was previously seen in the case of Jade Goody where the uptake in cervical screening was more pronounced in younger women and those from lower socio-economic backgrounds [21]. This current study did not detect a change in the socio-economic background of *Drink Less* users, based on employment type, suggesting that celebrity influence of Adrian Chiles did not negatively affect occupational inequalities in the use of *Drink Less*. However, the change in the sex and mean age of people downloading *Drink Less* was not sustained in the long-term. Therefore, ongoing media coverage may be needed if a sustained change in the characteristics of users is desired.

The media coverage of *Drink Less* also resulted in people with lower AUDIT scores downloading the app, though the proportion of at-risk drinkers did not change. Therefore, those people in need of support and who would benefit from reducing their drinking, but who were at the lower end of the at-risk scale appeared to have been influenced to download and use the app. This may be due to the narrative around Adrian Chiles’ TV documentary about how people’s alcohol consumption could still be problematic even if one is not, or does not see oneself as, dependent on alcohol. The documentary highlighted the issue of ‘othering’ – whereby people tend to perceive their own drinking as non-problematic because of a false binary surrounding ‘alcoholism’ [22]. Therefore, the media coverage may have influenced people who were hazardous drinkers to seek out digital support - rather than harmful drinkers or those with possible dependence, who represent a smaller proportion of the overall proportion of at-risk drinkers [23].

The number of sessions with *Drink Less* and the number of days it was used increased after the media coverage though no effect was detected on the time spent on the app or the proportion of screens viewed. These findings suggest that the media coverage and the recommendation from Adrian Chiles to use the app to track one’s drinking for  three weeks, did result in new users engaging with the app more frequently – both in terms of the number of sessions and days used – perhaps to track their drinking as suggested. This may also explain why no effect was detected on the time spent on the app or the proportion of screens viewed as tracking drinks tends to be a quick process involving a minimal number of screens. If drink tracking occurred regularly, it would result in an increase in the number of sessions and days used. The app reached a different and much larger group after the media coverage and whilst the new users did not use the app for longer or more deeply, it may still have had a positive impact on public health by increasing the reach of the app. It is also unclear whether a different message (e.g. to engage with all the app’s available features) may lead to increased time spent or screens viewed – this is currently explored in a different study, specifically designed to optimise the push notifications in *Drink Less* [24].

It is likely that the change in who downloads an app and how they use it will differ depending on the socio-demographic characteristics of the source and the narrative surrounding the media coverage. Therefore, it is important for future research to use other opportunities for natural experiments to assess whether these findings generalise to other digital interventions (e.g. apps for smoking cessation or physical activity promotion), and if any effect is dependent on who promotes the intervention and/or in what way. Further to this, it would be interesting for future research to study how celebrity influence may impact the behaviour of existing users of an app.

### Strengths and limitations

A major strength of this study is that it was an opportunistic natural experiment with a large sample. It was the first study to assess the impact of celebrity influence and consequent national media coverage over time on who then downloads and uses a digital intervention for alcohol reduction.

The study had a number of limitations. The findings may not generalise to other digital interventions and are likely to differ depending on the celebrity. There was only a comparatively short time series available after the media coverage and before the app was optimised (changing much of the content of the app). Therefore, we cannot distinguish between the type of promotion that occurred after the initial media coverage on 21st August 2018 as this coverage led to other types of coverage and promotion that was not feasible to track. Furthermore, we did not use a validated measure of socioeconomic position such as social grade [25]. Another limitation is that there may have been an increase in the proportion of users who were downloading the app out of curiosity and potentially reporting their consumption inaccurately as a result of the national media coverage. However, only those users who reported they were ‘interested in drinking less’ (instead of ‘just browsing’) when asked why they were using the app were included in the analytic sample.

## Conclusions

National media coverage of the *Drink Less* app because of celebrity influence, from the TV and radio presenter Adrian Chiles, shifted the socio-demographic and drinking characteristics of who used the app, as well as how they used it. After the media coverage, there were more male users of *Drink Less* and users who were older – potentially identifying with Adrian Chiles – and these users also engaged to a greater extent with the app.

## Supplementary Information

**Additional file 1: Figure S1.** Number of *Drink Less* app downloads by week for study period.

**Additional file 2: Table S1.** Timeline of Drink Less app versions.

**Additional file 3: Table S2.** AIC values for the trend analysis.

**Additional file 4: Table S3.** Interpretation of the change in slope coefficients for the linear, quadratic and cubic trend models.

**Additional file 5: Table S4.** Results of the unadjusted, best fitting model for engagement and drinking characteristics (accounting for seasonality and autocorrelation).

**Additional file 6: Table S5.** Results of the linear trend models for each outcome variable (accounting for seasonality and autocorrelation).

## Data Availability

The dataset generated and analysed during the current study is available in the Open Science Framework repository, https://osf.io/w73ud/.

## Abbreviations

ACF: Autocorrelation function
AIC: Akaike Information Criterion
AR: Autoregressive
AUDIT: Alcohol Use Disorders Identification Test
GAMMs: Generalised Additive Mixed Models
ITT: Intention-to-treat
MA: Moving average
PACF: Partial autocorrelation function
SD: Standard deviation
