# Supplementary figures and images for: The impact of celebrity influence and national media coverage on users of an alcohol reduction app: a natural experiment
Source: BMC Public Health. 2021 Jan 6;21:30. doi: 10.1186/s12889-020-10011-0 (PMC7789329; doi:10.1186/s12889-020-10011-0)

**Supplementary Figure 1:** Number of downloads by week for study period


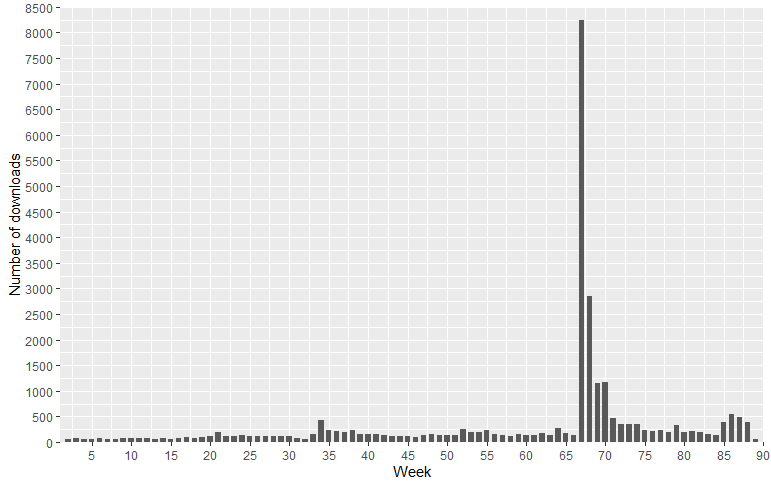

Supplement: Supplementary file 1 — Additional file 1: Figure S1. Number of Drink Less app downloads by week for study period. [file 12889_2020_10011_MOESM1_ESM.docx]
