# Supplementary material for: The impact of celebrity influence and national media coverage on users of an alcohol reduction app: a natural experiment
Source: BMC Public Health. 2021 Jan 6;21:30. doi: 10.1186/s12889-020-10011-0 (PMC7789329; doi:10.1186/s12889-020-10011-0)
Supplement: Supplementary file 2 — Additional file 2: Table S1. Timeline of Drink Less app versions. [file 12889_2020_10011_MOESM2_ESM.docx]

**Supplementary Table 1:** Timeline of Drink Less app versions

| *Drink Less* app version | Date of release | Release notes |
| --- | --- | --- |
| v1.0.11 | 17/5/2017 | Changes to onboarding procedure |
| v1.0.12 | 14/6/2017 | Fixes to minor bugs |
| v1.0.13 | 10/8/2017 | Minor updates and opt-out added to ‘Help’ section |
| v1.0.14 | 12/6/2018 | Bug fix for the calendar when the time zone changes |
| v1.0.15 | 23/7/2018 | Update to the Privacy Policy and drinking calendar changed so week starts on a Monday |
| v1.0.16 | 3/11/2018 | Fix for bug relating to clocks going backwards |
| v1.1.0 | 23/1/2019 | Changes to the content of the app: ability to update normative feedback and enter customisable drinks |
