# Supplementary material for: The impact of celebrity influence and national media coverage on users of an alcohol reduction app: a natural experiment
Source: BMC Public Health. 2021 Jan 6;21:30. doi: 10.1186/s12889-020-10011-0 (PMC7789329; doi:10.1186/s12889-020-10011-0)
Supplement: Supplementary file 3 — Additional file 3: Table S2. AIC values for the trend analysis. [file 12889_2020_10011_MOESM3_ESM.docx]

**Supplementary Table 2:** AIC values for the trend analysis

|  | Age | Sex | Employment type | AUDIT score | Percentage at-risk drinkers | Number of days used | Number of sessions | Percentage of screens viewed | Time on app | Percentage follow-up response | Reduction in past week alcohol consumption |
| --- | --- | --- | --- | --- | --- | --- | --- | --- | --- | --- | --- |
| AR and MA terms | AR(1), MA(0) | AR(2), MA(1) | AR(1), MA(0) | AR(0), MA(1) | AR(0), MA(1) | AR(1), MA(0) | AR(1), MA(0) | AR(1), MA(0) | AR(2), MA(1) | AR(1), MA(0) | AR(1), MA(1) |
| Linear trend | 306.6 | 573.2 | 519.7* | 201.4 | 439.5 | 256.1 | 369.0 | 306.1 | 572.6* | 461.1* | 236.5* |
| Quadratic trend | 307.4 | 574.1 | 519.8 | 199.3* | 437.6 | 248.3* | 365.4* | 304.8 | 572.4 | 462.2 | 238.3 |
| Cubic trend | 304.1* | 566.2* | 521.3 | 198.0 | 435.5* | 246.7 | 366.2 | 300.4* | 574.9 | 462.8 | 239.5 |

** best fitting model (model with lowest AIC value except if less than 2 AIC units, then simplest model)*
