# Supplementary material for: The impact of celebrity influence and national media coverage on users of an alcohol reduction app: a natural experiment
Source: BMC Public Health. 2021 Jan 6;21:30. doi: 10.1186/s12889-020-10011-0 (PMC7789329; doi:10.1186/s12889-020-10011-0)
Supplement: Supplementary file 4 — Additional file 4: Table S3. Interpretation of the change in slope coefficients for the linear, quadratic and cubic trend models. [file 12889_2020_10011_MOESM4_ESM.docx]

**Supplementary Table 3:** Interpretation of the change in slope coefficients for the linear, quadratic and cubic trend models

| Model | Coefficient | Interpretation |
| --- | --- | --- |
| Linear trend model | Change in slope | Linear slope between weeks since active promotion and the dependent variable. If the sign is positive then the dependent variable increases as time increases, if the sign is negative then the dependent variable decreases as time increases. |
| Quadratic trend model | Change in slope | Rate of change in dependent variable at the start of the series (i.e. active promotion begins). |
|  | Change in slope^2^ | The quadratic trend over the series. If the sign is positive then the model is convex (curvature is upwards) and if it is negative then curve is concave (curvature is downwards). |
| Cubic trend model | Change in slope | Rate of change in dependent variable at the start of the series (i.e. active promotion begins). |
|  | Change in slope^2^ | The quadratic trend over the series. If the sign is positive then the model is convex (curvature is upwards) and if it is negative then curve is concave (curvature is downwards). |
|  | Change in slope^3^ | The cubic trend over the series. If negative then the quadratic trend is increasingly negative as time increases. If positive then the quadratic trend is increasingly positive as time increases. |
