# Supplementary material for: The impact of celebrity influence and national media coverage on users of an alcohol reduction app: a natural experiment
Source: BMC Public Health. 2021 Jan 6;21:30. doi: 10.1186/s12889-020-10011-0 (PMC7789329; doi:10.1186/s12889-020-10011-0)
Supplement: Supplementary file 5 — Additional file 5: Table S4. Results of the unadjusted, best fitting model for engagement and drinking characteristics (accounting for seasonality and autocorrelation). [file 12889_2020_10011_MOESM5_ESM.docx]

**Supplementary Table 4:** Results of the unadjusted, best fitting model for engagement and drinking characteristics (accounting for seasonality and autocorrelation)

|  | Unadjusted | |
| --- | --- | --- |
|  | B (95% CI) | p |
| ***AUDIT score*** |  |  |
| *Quadratic model* |  |  |
| Trend | -0.02 (-0.03, -0.01) | <.001 |
| Change in slope | 0.32 (0.12, 0.52) | .003 |
| Change in slope^2^ | -0.01 (-0.02, -0.0001) | .043 |
| Level | -2.83 (-3.96, -1.69) | <.001 |
| ***Percentage at-risk drinkers*** |  |  |
| *Cubic model* |  |  |
| Trend | -0.01 (-0.04, 0.03) | .671 |
| Change in slope | 1.21 (-0.71, 3.13) | .219 |
| Change in slope^2^ | -0.15 (-0.34, 0.03) | .106 |
| Change in slope^3^ | 0.01 (0.00, 0.01) | .050 |
| Level | -5.56 (-11.11, -0.02) | .053 |
| ***Number of days used*** |  |  |
| *Quadratic model* |  |  |
| Trend | 0.01 (-0.01, 0.02) | .305 |
| Change in slope | -0.44 (-0.69, -0.19) | .001 |
| Change in slope^2^ | 0.02 (0.01, 0.03) | .001 |
| Level | 2.63 (1.23, 4.03) | <.001 |
| ***Number of sessions*** |  |  |
| *Quadratic model* |  |  |
| Trend | 0.01 (-0.02, 0.03) | .682 |
| Change in slope | -0.66 (-1.21, -0.11) | .021 |
| Change in slope^2^ | 0.03 (0.01, 0.05) | .019 |
| Level | 3.62 (0.63, 6.60) | .020 |
| ***Percentage of screens viewed*** |  |  |
| *Cubic model* |  |  |
| Trend | -0.02 (-0.04, -0.01) | .001 |
| Change in slope | 0.66 (-0.11, 1.42) | .095 |
| Change in slope^2^ | -0.08 (-0.15, -0.01) | .031 |
| Change in slope^3^ | 0.003 (0.001, 0.01) | .013 |
| Level | -1.76 (-4.00, 0.48) | .128 |
| ***Time on app*** |  |  |
| *Linear model* |  |  |
| Trend | -0.01 (-0.05, 0.04) | .748 |
| Change in slope | -0.18 (-0.52, 0.17) | .316 |
| Level | 0.20 (-5.34, 5.74) | .944 |
| ***Percentage follow-up response*** |  |  |
| *Linear model* |  |  |
| Trend | 0.03 (-0.01, 0.07) | .151 |
| Change in slope | -0.23 (-0.46, -0.002) | .051 |
| Level | 1.68 (-1.85, 5.20) | .353 |
| ***Reduction in past week alcohol consumption (ITT)*** |  |  |
| *Linear model* |  |  |
| Trend | 0.002 (-0.003, 0.007) | .430 |
| Change in slope | -0.02 (-0.06, 0.02) | .417 |
| Level | 0.17 (-0.46, 0.80) | .599 |
