# Supplementary material for: The impact of celebrity influence and national media coverage on users of an alcohol reduction app: a natural experiment
Source: BMC Public Health. 2021 Jan 6;21:30. doi: 10.1186/s12889-020-10011-0 (PMC7789329; doi:10.1186/s12889-020-10011-0)
Supplement: Supplementary file 6 — Additional file 6: Table S5. Results of the linear trend models for each outcome variable (accounting for seasonality and autocorrelation). [file 12889_2020_10011_MOESM6_ESM.docx]

**Supplementary Table 5:** Results of the linear trend models for each outcome variable (accounting for seasonality and autocorrelation)

|  | Unadjusted | | Adjusted | |
| --- | --- | --- | --- | --- |
|  | B (95% CI) | p | beta (95% CI) | p |
| ***Age*** |  |  |  |  |
| Trend | 0.03 (0.01, 0.05) | .019 | - | - |
| Change in slope | -0.19 (-0.30, -0.07) | .002 | - | - |
| Level | 4.89 (3.12, 6.65) | <.001 | - | - |
| ***Sex (% female)*** |  |  |  |  |
| Trend | -0.04 (-0.07, -0.01) | .006 | - | - |
| Change in slope | 0.28 (0.003, 0.56) | .051 | - | - |
| Level | -6.05 (-10.04, -2.07) | .004 | - | - |
| ***Employment type (% non-manual)*** |  |  |  |  |
| Trend | -0.01 (-0.06, 0.04) | .712 | - | - |
| Change in slope | -0.11 (-0.34, 0.13) | .387 | - | - |
| Level | 3.14 (-0.54, 6.82) | .098 | - | - |
| ***AUDIT score^a^*** |  |  |  |  |
| Trend | -0.02 (-0.03, -0.01) | <.001 | -0.01 (-0.02, -0.004) | .009 |
| Change in slope | 0.12 (0.06, 0.17) | <.001 | 0.07 (0.01, 0.13) | .021 |
| Level | -2.07 (-2.97, -1.18) | <.001 | -0.84 (-1.82, 0.14) | .097 |
| ***Percentage at-risk drinkers^a^*** |  |  |  |  |
| Trend | -0.01 (-0.05, 0.03) | .744 | 0.01 (-0.04, 0.05) | .820 |
| Change in slope | 0.16 (-0.05, 0.37) | .142 | 0.08 (-0.14, 0.31) | .467 |
| Level | -4.88 (-8.19, -1.58) | .005 | -2.96 (-6.85, 0.92) | .139 |
| ***Number of days used^a^*** |  |  |  |  |
| Trend | 0.01 (-0.01, 0.02) | .382 | 0.003 (-0.01, 0.02) | .646 |
| Change in slope | -0.004 (-0.08, 0.07) | .919 | -0.002 (-0.07, 0.07) | .956 |
| Level | 0.81 (-0.29, 1.91) | .153 | 0.40 (-0.81, 1.61) | .516 |
| ***Number of sessions^a^*** |  |  |  |  |
| Trend | 0.01 (-0.03, 0.04) | .763 | 0.002 (-0.03, 0.03) | .921 |
| Change in slope | -0.01 (-0.16, 0.14) | .890 | -0.02 (-0.17, 0.13) | .800 |
| Level | 1.07 (-1.20, 3.34) | .358 | 0.79 (-1.71, 3.29) | .536 |
| ***Percentage of screens viewed^a^*** |  |  |  |  |
| Trend | -0.02 (-0.04, -0.01) | .006 | -0.02 (-0.04, -0.01) | .009 |
| Change in slope | 0.02 (-0.06, 0.11) | .554 | 0.01 (-0.08, 0.09) | .881 |
| Level | -1.06 (-2.33, 0.21) | .105 | -0.74 (-2.30, 0.82) | .357 |
| ***Time on app^a^*** |  |  |  |  |
| Trend | -0.01 (-0.05, 0.04) | .748 | 0.004 (-0.04, 0.05) | .849 |
| Change in slope | -0.18 (-0.52, 0.17) | .316 | -0.03 (-0.35, 0.29) | .833 |
| Level | 0.20 (-5.34, 5.74) | .944 | -4.14 (-9.15, 0.88) | .110 |
| ***Percentage follow-up response^a^*** |  |  |  |  |
| Trend | 0.03 (-0.01, 0.07) | .151 | 0.02 (-0.03, 0.06) | .449 |
| Change in slope | -0.23 (-0.46, -0.002) | .051 | -0.10 (-0.32, 0.11) | .356 |
| Level | 1.68 (-1.85, 5.20) | .353 | -1.57 (-5.34, 2.21) | .418 |
| ***Reduction in past week alcohol consumption (ITT)^b^*** |  |  |  |  |
| Trend | 0.002 (-0.003, 0.007) | .430 | 0.004 (-0.003, 0.01) | .288 |
| Change in slope | -0.02 (-0.06, 0.02) | .417 | -0.02 (-0.06, 0.02) | .374 |
| Level | 0.17 (-0.46, 0.80) | .599 | 0.23 (-0.43, 0.89) | .489 |

^a^ adjusted for socio-demographic characteristics
^b^ adjusted for baseline AUDIT scores
